# Porosity Formation in Thin Welded Joints of Al–MG–LI Alloys

**DOI:** 10.3390/ma15010348

**Published:** 2022-01-04

**Authors:** Tatyana Olshanskaya, Elena Fedoseeva

**Affiliations:** Department of Welding Production, Metrology and Technology of Materials, Perm National Research Polytechnic University, 614990 Perm, Russia; elena.fedoseeva.79@mail.ru

**Keywords:** aluminum alloy, pore formation, thermal vacuum treatment, welded joint, welding technology, thermodynamic calculation, microhardness, thermal cycle

## Abstract

This work is about the study of the correlation of pore formation in welded joints of Al–MG–LI alloy with zirconium additives with the state of the base metal, thermal vacuum treatment, and welding technologies MIG and EBW. Metallographic analysis has been carried out, the phase composition of the alloy and weld metal has been investigated, and thermal cycles of welding have been calculated, allowing to estimate the residence time of metal in the alloying zone and weld metal in the liquid state. The nature of the allocation of strengthening fine-dispersed phases in the welded joints of the alloy has been determined. The regularity and character of pore formation in welded joints depending on the applicable thermal vacuum treatment (TVT) and welding technology have been revealed. It was established that TVT with subsequent hardening and aging has no effect on the phase composition of the alloy. However, this type of treatment contributes to the formation of a more homogeneous and uniform nature of the separation of fine-dispersed strengthening phases. It was revealed that the MIG technology (metal with and without TVT) is characterized by a large length of the fusion zone, the high residence time of metal in the fusion zone and weld metal in the liquid state, and the formation of pores. Phase formation in the temperature range of the beginning and end of the alloy crystallization occurs not only in the weld at the final stage of crystallization but also in the fusion zone, which may induce pore formation, whereas EBW welding shows the opposite trend and no pores. It was found that EBW technology prevents pore formation and makes it possible to obtain welded joints of 1420 Al alloys of the required quality.

## 1. Introduction

Aluminum alloys are known for their variety of classification by alloying elements that provide some properties to the alloys, and research is quite actual. Al–MG–LI alloys with zirconium additives are used in the aerospace industry and other areas of production due to their unique properties. For every 1% of lithium, the density of the alloy decreases by 3%, while Young’s modulus increases by 5%. The modulus of elasticity of aluminum, magnesium, and lithium are 7100, 4300, and 500 kg/mm^2^, respectively [1,2,3,4,5].

Al–MG–LI alloys, despite the extremely low corresponding lithium characteristic, have a significantly higher modulus of elasticity, consequently, these alloys have an exceptionally high modulus of elasticity [6,7]. Due to the combination of low density, high elastic modulus, high corrosion resistance, and weldability, Al–MG–LI alloys are important in industry and are widely used in the manufacture of lightweight structures of small thickness, in particular aircraft structures [8,9,10]. The Al–MG–LI alloys include the 1420 deformation alloy with a density of 2.47 g/sm^3^, and it is the lightest among aluminum alloys [11].

Both the most efficient and promising technological process for the production of monolithic components of various structures are welding. The weldability of 1420 aluminum alloy has several features associated with the physical, chemical, and technological properties of the material [12,13,14,15]. The main difficulties are caused by the presence of a strong oxide layer, which has a high adsorption capacity concerning gases, and which greatly distinguishes almost all aluminum alloys from steel.

The moisture adsorbed on the porous oxide film in the form of variable aluminum hydroxide (Al_2_O_3_·nH_2_O) and other impurities interact with the liquid metal of the weld pool and release hydrogen gas bubbles into the molten metal. Aluminum, which is characterized by high chemical activity, easily interacts with air oxygen and forms an oxide film [16,17].

One of the main sources of pore formation for Al–MG–LI alloys is considered to be the surface layer formed during manufacturing and process heating of parts because the surface layer compounds dissociate during welding heating with the release of gases. In this regard, 1420 alloy in thin welding joints in the aviation industry have led to the formation of porosity caused by presumably high hydrogen concentration in the base material.

The All-Russian Scientific Research Institute of Aviation Materials (VIAM) recommended, as one of the solutions to prevent porosity in welds of Al–MG–LI alloys in thin welded constructions in the aviation industry, the use of the thermal vacuum treatment of material before welding. Thermal vacuum treatment is applied to change the composition of joints and reduce the concentration of hydrogen in the base metal, which will improve the quality of the initial material, reduce the amount of released hydrogen, and as a consequence, reduce pore formation.

Hydrogen is the main cause of porosity in welding aluminum alloys. In the liquid state, aluminum dissolves hydrogen quite easily, which is due to the high rates of effective diffusion of hydrogen in liquid aluminum [1]. The solubility of hydrogen in aluminum decreases during the transition from liquid to solid state; this leads to the formation of porosity. It has been proven [12] that the gases in the pores have a complex composition, and hydrogen plays a decisive role here. When welding aluminum, larger crystals grow from the surface of melted grains by one or two orders of magnitude across the cross-section, at the joints and ends of which recesses are created that facilitate the nucleation of gas bubbles.

The pore-forming hydrogen can enter the weld pool not only from the metal that is welded but also from the welding materials and shielding gas [18]. The right choice of welding technology for Al alloys allows obtaining welded joints without defects and pores. In this case, both arc welding methods (argon arc welding) and welding technology with highly concentrated energy sources (electron beam welding) are applicable [18]. Both of them have certain characteristics. The accumulated information on the welding of Al–MG–LI alloys shows that automatic argon arc welding with alternating current is widely used for welding products of thin welded joints in aircraft construction. It is necessary to take into account that one of the important factors influencing the formation of quality welded joints is the humidity of shielding gas, which should be controlled. In addition, it is necessary to improve the protection of the weld root from oxidation and to perform chemical polishing of the welding wire [19,20,21].

Electron beam welding (EBW) is an alternative method for welding Al alloys of thin joints, which provides better weld quality. EBW is carried out by a highly concentrated energy source, in a vacuum chamber, which provides good degassing of the metal, and without the use of welding materials (welding wire and shielding gas).

Although earlier studies of Al–MG–LI alloys welded joints have been conducted, the interaction between pore formation, thermal vacuum treatment, and welding technologies of Al–MG–LI alloys has not been investigated. Currently, a comparison between EBW and MIG welding of 1420 Al alloy is missing within the scientific literature. This study is aimed at substantiating the choice of welding technology in thin welded joints of Al–MG–LI alloys. The influence of base material, thermal vacuum treatment, and welding technologies on pore formation has been analyzed in welded joints of Al–MG–LI alloys.

## 2. Materials and Methods

Two welding technologies were used to obtain experimental blanks with a thickness of 3 mm made of 1420 Al–MG–LI alloy with zirconium. Welding of thin plates of 1420 alloy with the different initial states was carried out by automatic argon arc welding (MIG) with an additive wire AlMg-6 and by electron beam welding (EBW). The average chemical composition of AMg-6 welding wire is shown in Table 1.

Part of the samples before welding, after standard heat treatment T6, were subjected to thermal vacuum treatment (TVT) according to the following modes [13,14,15,16]: (1) Thermal vacuum treatment at temperature (450 ± 10 °C) for 6 h, and vacuum not lower than 1 × 10^−3^ mm Hg. Processing time is assigned depending on the thickness of the material. Cooling with furnace up to 80 °C, then in the air. (2) Followed by standard heat treatment T6. Thermal vacuum treatment of 1420 alloy workpieces was performed in the electric resistance chamber furnace of vacuum model ESCVE-16/16GM1 (“Research and Production Enterprise MosZETO”, Moscow, Russia).

Keller reagent (hydrofluoric acid HF 2 mL 48%, hydrochloric acid HCl 3 mL, water H2O 190 mL) was used to reveal the microstructure on the sections. The research was conducted on an Altami CM0745-T optical stereomicroscope (Altami, Saint Petersburg, Russia) and an Altami MET 1T inverted light microscope (Altami, Saint Petersburg, Russia) with a magnification of up to 1000 times using Altami Studio 3.5 software (version 3.5, Altami, Saint Petersburg, Russia). Hardness is measured by the Vickers method on microhardness PMT-3 at a load of 50 g. The microhardness was carried out along the welded joint along with the following directions: base metal, heat affected zone, welded seam, heat affected zone, base metal.

Analysis of thermodynamic calculations of possible phases during equilibrium crystallization of the alloy (in coordinates of % phases from temperature) was made using the JMatPro the Materials Property Simulation Package the company Sente Software Ltd. (Surrey, UK). JmatPro is a simulation software which calculates a wide range of materials properties for alloys and is particularly aimed at multi-component alloys used in industrial practice. Using JmatPro allows calculations for: stable and metastable phase equilibria, solidification behavior and properties, mechanical properties, thermo-physical and physical properties, phase transformations, and chemical properties.

The phase composition of the samples was studied using X-ray diffractometer XRD-7000. A monochromator was used to separate the Kβ component of the X-ray radiation during the analysis. Imaging conditions: scanning angle range 2 Theta from 30 to 90, X-ray tube—Cu, tube voltage 30 kV, tube current 30 mA, scanning speed 1 deg/min, scanning step 0.01 deg, slits DS 1 deg, SS 1 deg, RS 0.15 mm. Processing of X-ray images was performed using the software PANalytical X’Pert HighScore Plus Ver.2.1 (version 2.1, PANalytical, 2004, Almelo, The Netherlands). Determination of the phase composition of the analyzed samples was performed using the database “ICDD PDF-2+ 2012”.

## 3. Results

### 3.1. Calculated and Experimental Analysis of the Influence of TVT on the Structural and Phase Composition of the 1420 Alloy

Calculated analysis of the Al–MG–LI alloy the phase composition after the standard heat treatment of the deformed metal and after TVT was carried out using the JMatPro program on the example of 1420 alloy. For the calculation, the average value of the main alloying elements was taken in accordance with OST 1-90048-90-90 (Table 2).

**Figure 1:**
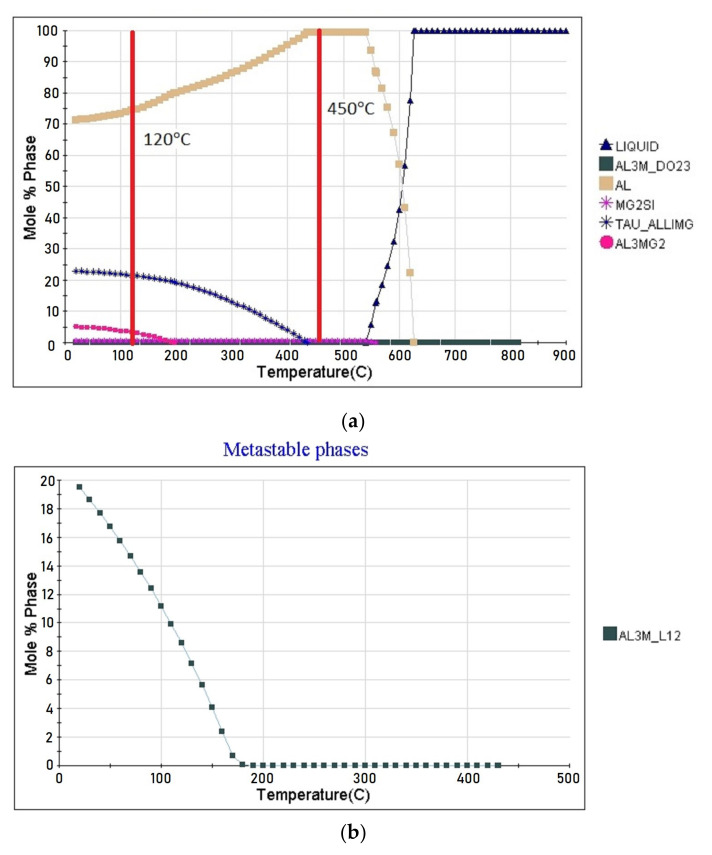
Thermodynamic calculation of the alloy the phase composition of: (**a**) stable phases; (**b**) metastable phases.

Figure 1a shows the result of the thermodynamic calculation of the alloy the phase composition as a function of temperature. The calculation was performed in the temperature range from 900 to 20 °C in 10 °C steps. The calculations showed that the equilibrium temperatures of the beginning and the end of crystallization for the alloy are 627 and 540 °C, respectively. The following stable strengthening phases can form in the alloy (in parentheses is the phase designation in the software package JMatPro):

Al_3_Zr (AL3M_DO23)—formed at 815 °C in the liquid alloy; Mg_2_Si (MG2SI)—formed at 559 °C in the temperature range of the beginning and end of crystallization of the solid solution based on aluminum; Al_2_LiMg (TAU_ALLIMG)—begins to be released at 435 °C from the solid solution; Al_3_Mg_2_ (AL3MG2)—begins to be released at 190 °C from the solid solution.

The concentration of chemical elements in these phases varies insignificantly depending on the temperature. A metastable phase Al_3_Li (AL3M_L12) may be in the alloy at temperatures lower than 190 °C in addition to the stable phases (Figure 1b). Its content and chemical composition also depend on the temperature.

**Figure 2:**
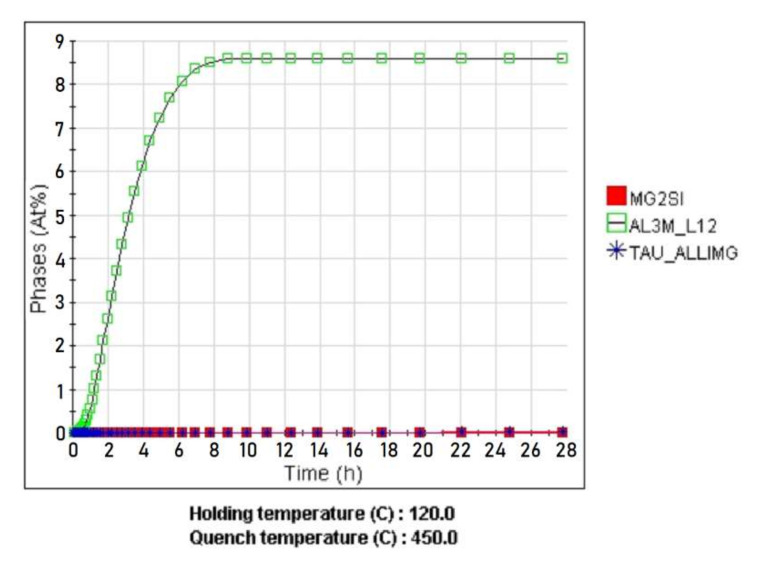
Calculation of the allocation of metastable phases during aging.

During the quenching process at 450 °C, the hardening phases Al_2_LiMg (TAU_ALLIMG and Al_3_Mg_2_ (AL3MG2) dissolve. After quenching, the alloy consists completely of solid solution-based aluminum, less than 1% of the non-dissolved phases Al_3_Zr (AL3M_DO23) and Mg_2_Si (MG2SI). As a result of artificial aging at 120 °C, the hardening stable phases Al_2_LiMg (TAU_ALLIMG) and Al_3_Mg_2_ (AL3MG2) and the metastable phase Al_3_Li (AL3M_L12) are released; the calculation of the release of this phase is shown in Figure 2. Table 3 shows the results of the calculation of the alloy phase composition and the chemical composition of the strengthening phases after the T6 heat treatment.

Thus, according to the calculated results, the main strengthening phases of 1420 alloy after T6 heat treatment will be Al_2_LiMg (TAU_ALLIMG), Al_3_Mg_2_ (AL3MG2), Al_3_Li (AL3M_L12).

**Figure 3:**
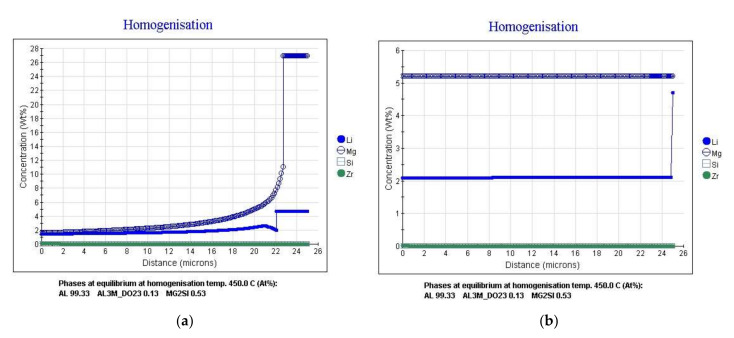
The distribution of chemical elements in the grain of the α-solid solution after different durations of exposure at 450 °C: (**a**) 30 min; (**b**) 6 h (TVT).

**Figure 4:**
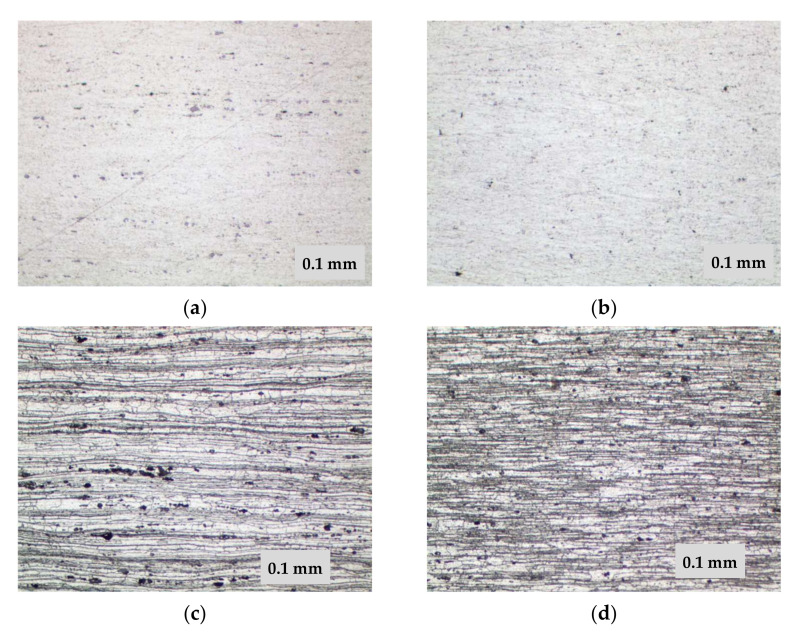
Microstructure of 1420 alloy after standard heat treatment (**a**,**c**) and after TVT (**b**,**d**), ×200: (**a**,**b**) not etched surface; (**c**,**d**) after etching.

Calculation of the thermal vacuum treatment simulation of the alloy showed that in the process of exposure at 450 °C for 6 h there is a significant alignment of chemical elements in the body of the grain (Figure 3). The homogenization process leads to the fixation of a homogeneous solid solution during subsequent hardening. This contributes to a uniform release of fine-dispersed strengthening phases during aging, which is confirmed by the results of metallographic analysis of the microstructure of the alloy (Figure 4).

**Figure 5:**
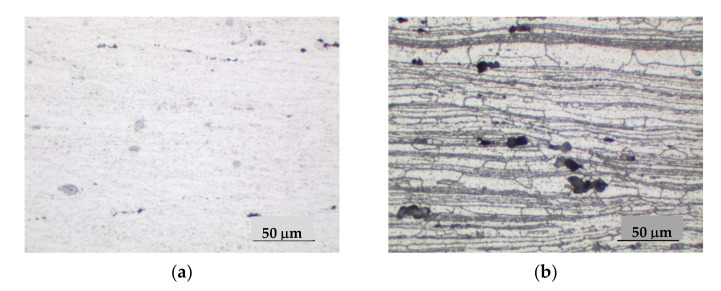

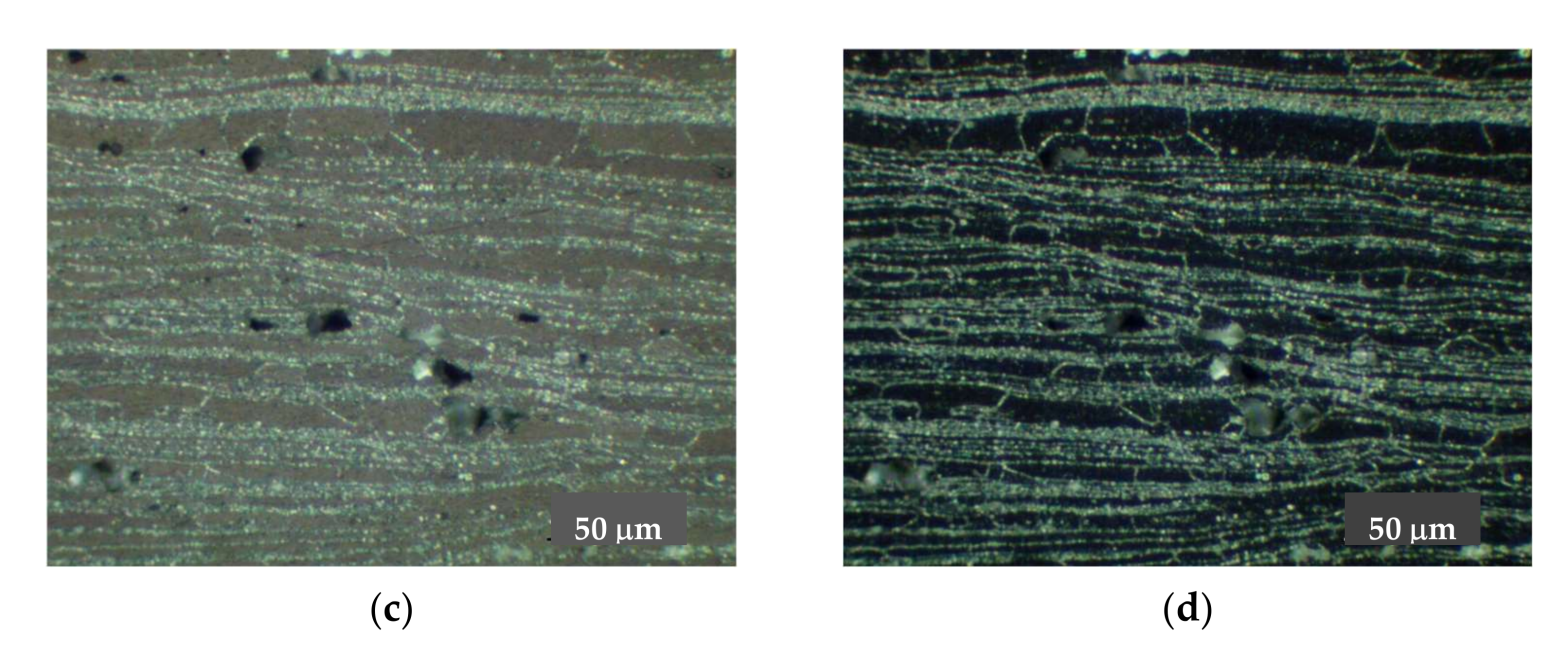
Microstructure of 1420 alloy after standard heat treatment, ×500: (**a**) not etched surface; (**b**–**d**) after etching; (**b**) light field; (**c**) polarized light; (**d**) dark field.

Metallographic analysis of the 1420 alloy metal (not previously subjected to TVT) showed a characteristic allocation of hardening phases of different shapes as shown in Figure 4a and Figure 5a. The distribution of strengthening phases in the studied areas is heterogeneous. Some are in the form of chains. Investigations in the dark field allowed the identification of pores. As can be seen in Figure 5c,d, they are characterized by the luminous contour appearance. In addition, in polarized light with gradual quenching, the presence of small pores dispersed across the cross-section of the metal was found. Analysis of the alloy metal after etching showed a pronounced structure of the material with traces of deformation texture as seen in Figure 4c and Figure 5b–d.

**Figure 6:**
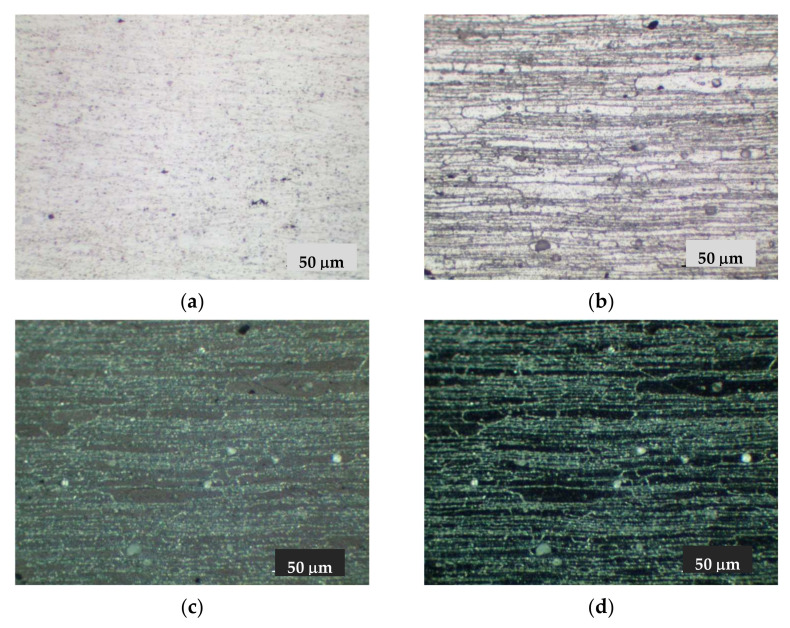
Microstructure of 1420 alloy after TVT, ×500: (**a**) not etched surface; (**b**–**d**) after etching; (**b**) light field, (**c**) polarized light, (**d**) dark field.

The metallographic studies of the alloy metal subjected to TVT were obtained as follows. In the alloy metal, there are both strengthening phases and a number of pores (Figure 4b, Figure 5d and Figure 6a,d). However, the nature of the distribution of strengthening phases is more uniform. The traces of deformation texture have not disappeared completely, which should disappear under the condition of thermal vacuum treatment because at the given temperatures theoretically recrystallization process takes place and the grains acquire an equiaxed appearance. However, as we can see in Figure 4d and Figure 6b, the deformation texture traces are smoothed and the structure becomes more uniform. In addition, the formation of strengthening phases is also more uniform (Figure 4d), in contrast to the metal without TVT. Figure 6c,d shows the nature of distribution and allocation of strengthening phases and some presence of pores.

**Figure 7:**
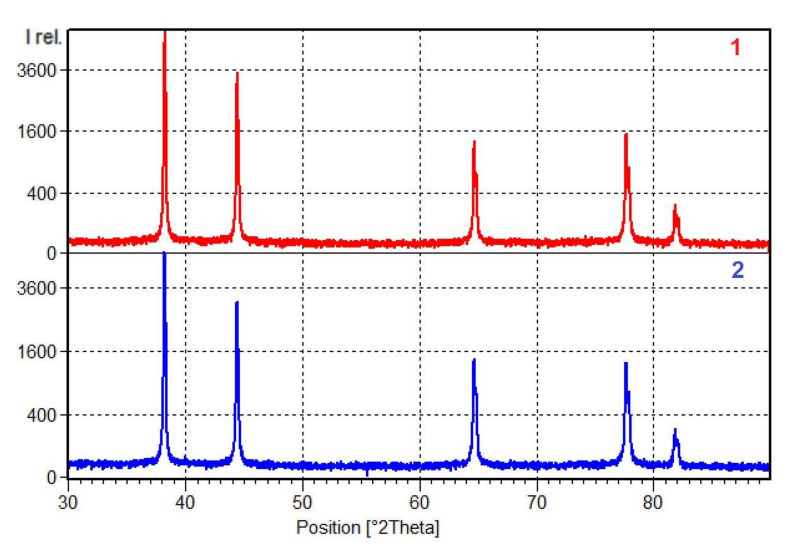
Diffractograms for the 1420 alloy after standard heat treatment T6 (**1**) and after TVT (**2**).

**Figure 8:**
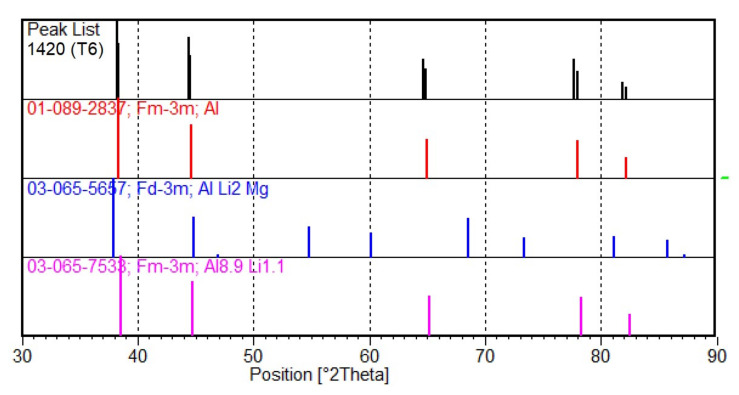
Results of decoding of diffraction patterns.

The calculations also showed that TVT with subsequent quenching and aging has no effect on the phase composition of the alloy. The calculations are confirmed by the results of qualitative X-ray phase analysis. Figure 7 shows the diffractograms for the alloy after standard heat treatment T6 (1) and after TVT (2), which almost completely coincide with each other. The results of the X-ray phase analysis are presented in Figure 8 and Table 4.

The peaks in the diffractograms of the alloy are characteristic of the following compounds:

- Al, Cubic, spatial group Fm-3m (225) (№ 01-089-2837 in the database);
- Aluminum Lithium Magnesium AlLi_2_Mg, Cubic, spatial group Fd-3m (227) (№ 03-065-5657 in the database);
- Aluminum Lithium AL_8.9_Li_1.1_, Cubic, spatial group Fm-3m (225) (№ 03-065-7533 in the database).

The chemical composition of the substance number 00-026-0042 is close to the calculated phase Al_2_LiMg (TAU_ALLIMG), and the substance 03-065-7533 is close to the phase Al_3_Li (AL3M_L12). Other calculated phases with content less than 5% are practically not determined by the X-ray phase analysis.

### 3.2. Investigation of the TVT Influence and Welding Method on the Formation of Welded Joints of the 1420 Alloy Al–MG–LI Alloying System

**Figure 9:**
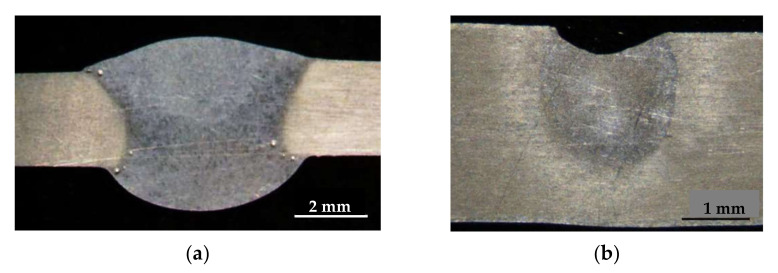
Macrostructure of welds made by different welding methods of 1420 alloy at different initial state: (**a**) argon arc welding (MIG) with AlMg-6 wire, base material after TVT; (**b**) electron beam welding (EBW), base material after TVT.

The macrostructure investigation of welded joints was carried out on samples cut from the cross-section. The analysis of macrostructure studies showed that in the process of MIG welding pore formation is observed in the welded joints regardless of the initial state of the base metal. Pores are formed mainly at the boundary between the weld and the base metal. In the case of alloy welding after TVT, the pores are also present at the boundary between the layers (Figure 9). However, no pore formation was observed in the welded joints by EBW, regardless of the initial state of the alloy (Figure 9).

#### 3.2.1. Study of the Microstructure of the Welded Joint Made by MIG Welding

**Figure 10:**
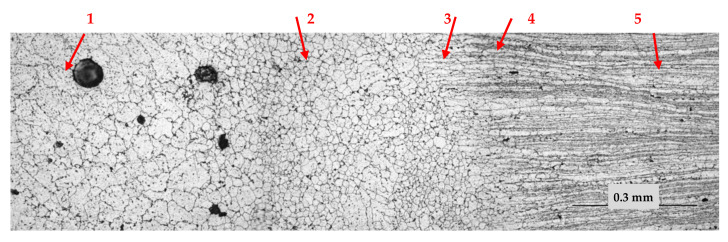
Structure of the welded joint made by MIG, base metal after TVT, ×200; 1—weld, 2—fusion zone, 3—superheat zone, 4—aging zone, 5—base metal.

**Figure 11:**
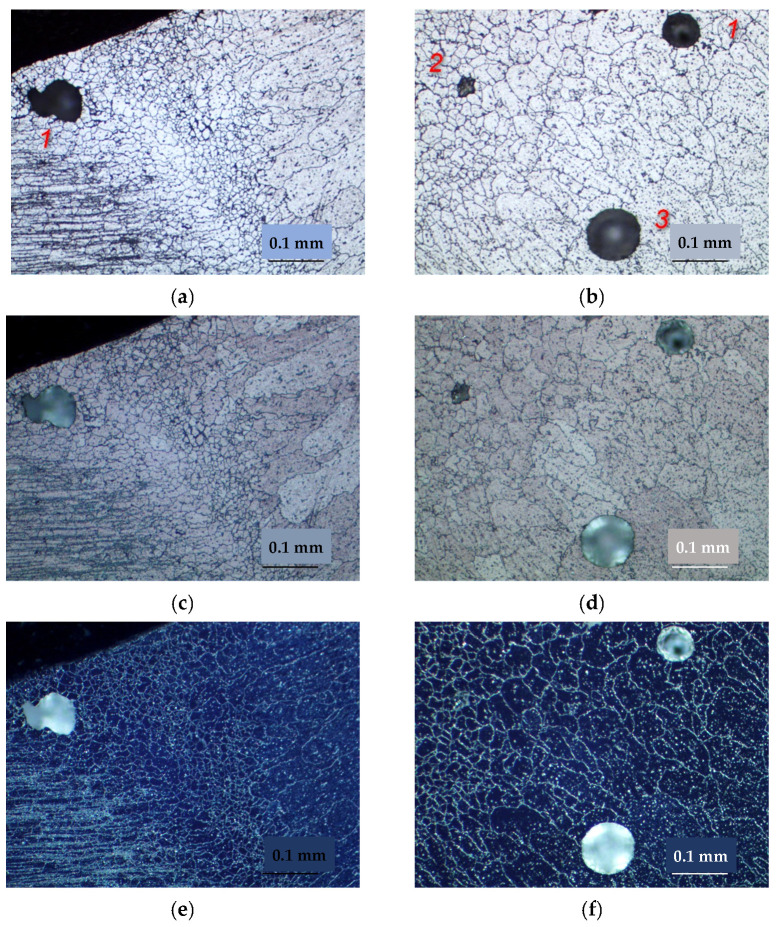
Defect formation in the fusion zone, ×200: 1—pores, 2—large inclusions, 3—silicate (SiO2); (**a**,**b**) light field; (**c**,**d**) polarized light; (**e**,**f**) dark field; (**a**,**c**,**e**) basic material after T6 heat treatment; (**b**,**d**,**f**) basic material after TVT.

**Figure 12:**
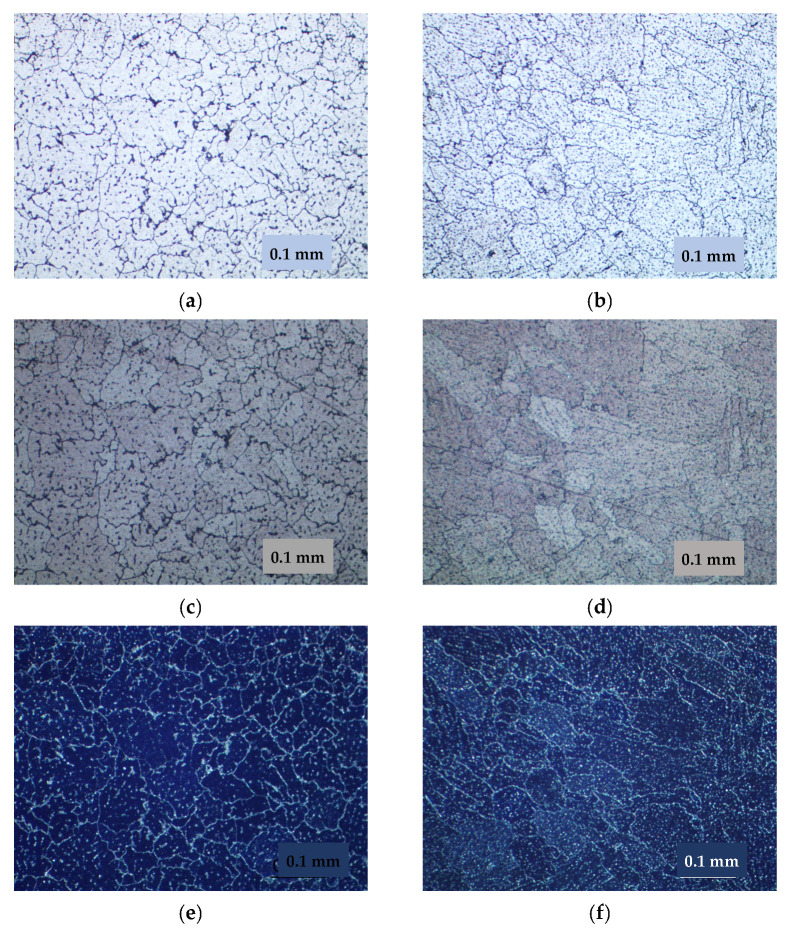
Microstructure of the weld metal, ×200: (**a**,**b**) light field; (**c**,**d**) polarized light; (**e**,**f**) dark field; (**a**,**c**,**e**) basic material after T6 heat treatment; (**b**,**d**,**f**) basic material after TVT.

The weld joint consists of the weld metal, the fusion zone, the superheat zone, where grain growth and dissolution of strengthening phases are observed, and the aging zone, where phase coagulation is observed. The fusion zone is characterized by a large extent to which a layer of fine equiaxed grains is formed (Figure 10). Studies have shown that pore formation occurs directly in the fusion zone (Figure 11), regardless of the state of the base metal. At welding of the alloy after the TVT in the fusion zone, in addition to pores, the formation of large inclusions, up to silicates (SiO_2_) is observed. Determination of the type of inclusions and pores was carried out using polarized light and temperature field illumination on an optical microscope. Volumetric and anisotropic phases such as intermetallic inclusions “glow” in the dark field, in polarized light they acquire a certain color and change their illumination. Silicate inclusions have a regular circular shape, in the light field concentric rings appear, and in the dark field and polarized light an optical cross effect is observed. Spherical pores in the volume of the material appear as dark spots, in the dark field and in polarized light a specific light figure is formed, the sharpness of which depends on the depth of occurrence [22]. The weld metal is characterized by the formation of equiaxed grains with the allocation of strengthening phases both within the grain and on the borders. In the metal of the weld, it can also be observed the allocation of single pores and clusters of large inclusions, including silicates (SiO_2_) (Figure 12).

Table 5 welding wire is magnesium and small amounts of manganese, titanium, and silicon (Table 1). The alloy of this composition refers to thermally unstable; thus, there are no metastable phases in it. Respectively, the phase composition of the weld metal will differ from the base metal.

**Figure 13:**
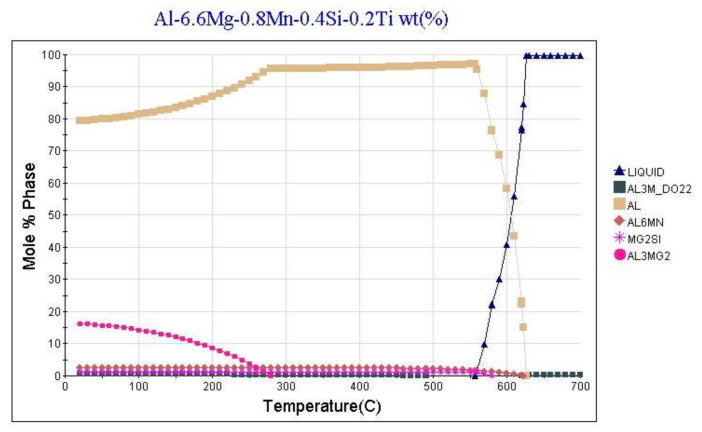
Thermodynamic calculation of weld metal phase composition with AlMg-6 welding additive wire.

Thermodynamic calculation of changes in the phase composition of the alloy as a function of temperature was made in the program JMatPro for the weld metal. The results are presented in Table 5 and Figure 13. The calculations showed that during crystallization the following strengthening phases will form in the weld metal: Al_3_Ti (AL3M_DO22), Al_6_Mn (AL6MN), Mg_2_Si (MG2SI), Al_3_Mg_2_ (AL3MG2). In this case, the two main phases (Al_6_Mn (AL6MN) and Al_3_Mg_2_ (AL3MG2)) are formed in the temperature range of the beginning and end of crystallization of the alloy. That is, these phases are formed not only in the weld at the final crystallization stage but also in the fusion zone. The formation of phases in the two-phase solid–liquid fusion zone can also initiate the formation of pores. The third hardening phase (Al_3_Mg_2_ (AL3MG2)) is formed at temperatures below 280 °C and is released inside the grain.

Therefore, the use of TVT by MIG welding does not allow to improve the quality of welded joints to reduce pore formation.

#### 3.2.2. Research on the Microstructure of a Welded Joint Made by the EBW

**Figure 14:**
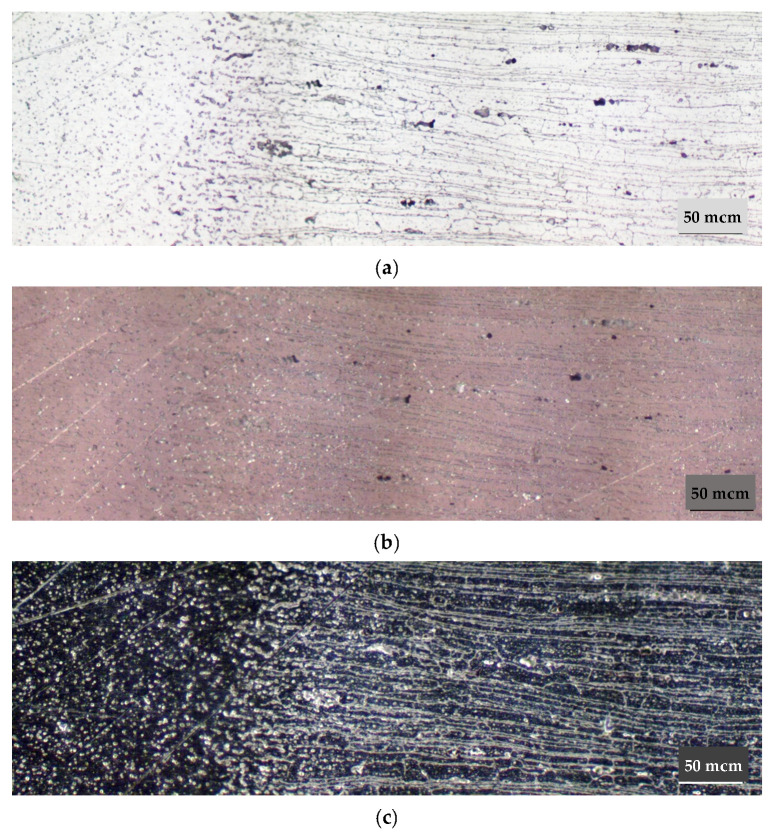
Structure of the welded joint made by the EBW, 500: (**a**) light field; (**b**) polarized light; (**c**) dark field.

**Figure 15:**
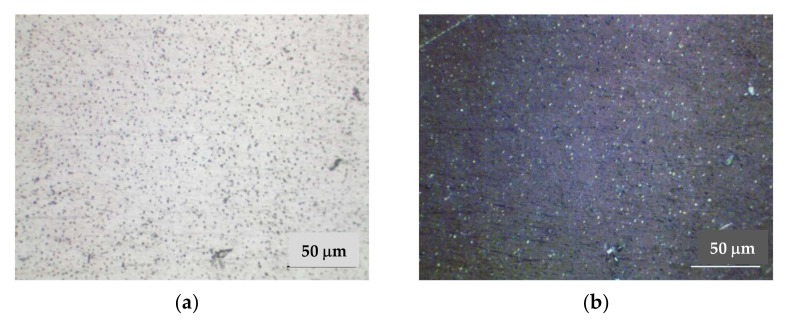
Microstructure of the weld metal made by EBW, ×500: (**a**) light field; (**b**) polarized light.

EBW was carried out without additive material; thus, the metal chemical and phase composition will correspond to the base metal. The extent of the fusion zone in the heat-affected zone is considerably less in the welded joints made by the EBW than by the MIG welding. The fusion zone consists of 1–2 rows of fine equiaxed grains (Figure 14). The overheat area is almost absent, and the aging area increases. Strengthening phases are evenly distributed in the weld metal without etching. It was not possible to identify grain boundaries during etching, uniformly distributed etch pits formed around the finely dispersed phases are evident (Figure 15). This indicates that the formation of a homogeneous solid solution occurred during the crystallization of the weld metal. On subsequent cooling, the hardening phase of the Al_3_Mg_2_ was released.

**Figure 16:**
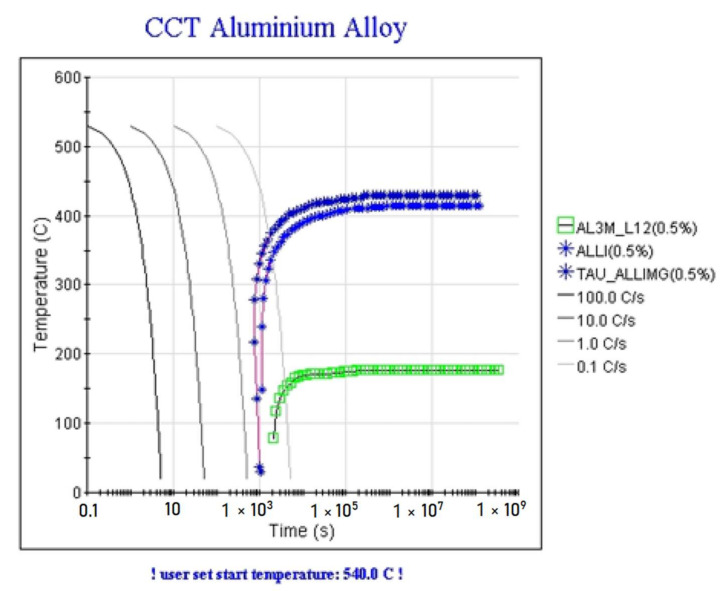
Calculated cooling time–temperature–transformational diagram (CCT) for 1420 alloy.

Calculation of the cooling time-temperature-transformational diagram was carried for 1420 alloy from 540 °C close to the temperature of the end of crystallization out to analyze the forming structure in the weld. According to the diagram shown in Figure 16, a supersaturated solid solution is formed at cooling rates greater than 10 °C/s.

### 3.3. Microhardness Results

**Figure 17:**
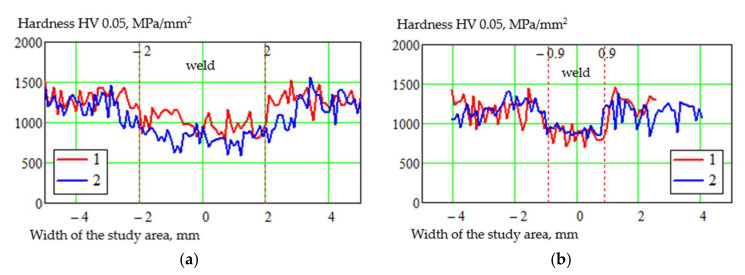
Distribution of microhardness in the cross-section of samples at different initial state: 1— without TVT, 2—with TVT; (**a**) MIG; (**b**) EBW.

The microhardness has given the following results (Figure 17 and Table 6).

As we can see from Figure 17, the welds show a decrease in hardness relative to the base material, in all cases. It is since a cast structure is formed in the weld. The use of TVT before welding results in a more homogeneous hardness of the weld metal in both welding cases.

The hardness of the base material decreases more concerning the base metal when welded after TVT.

### 3.4. Calculated Analysis of Temperature-Time Parameters during Welding of 1420 Alloy

The formation of the welded joints structure, as well as the formation of pores directly depends on the temperature-time parameters of the welding process. To evaluate and compare the thermal cycles of arc and electron beam welding of thin welded joints of 1420 alloys, computational studies were carried out using a thermal model. A model of heating a semi-infinite plate by a normal-circular source, based on the differential equation of heat conduction in a moving coordinate system with a stationary source, and obtained by the analytical method using Green’s functions, was used for calculation:

(1) $$T\left(x,y,z,t\right)=\frac{q\eta }{4c\rho {\sqrt{a\pi }}^{3}}\overset{t}{\underset{t0}{\int }}\frac{1}{{\sqrt{\tau }}^{3}}\cdot exp\left[-\frac{{\left(x+V\tau \right)}^{2}+y^{2}}{4a\pi }\right]\cdot \overset{\infty }{\underset{-\infty }{\sum }}exp\left[-\frac{{\left(z+2n\delta \right)}^{2}}{4a\pi }\right]d\tau$$

where *V*—welding speed, *δ*—thickness of the plate, *τ*—the times of the source, *q*—heat source power, *η*—coefficient of efficiency, *c*—heat conductivity, *ρ*—density of the metal, *t*0—operation time of the simulated heat source, depends on the concentration factor of the heat source.

The instant cooling rate was defined as:

(2) $$W=\frac{dT\left(x,y,z,t\right)}{dt}$$

Given that in welding there is a relationship between the distance of the reference point from the heat source and time (*dt* = *dx*/*V*, where *V* is the welding speed), Equation (2) takes the following form:

(3) $$W=\frac{dT\left(x,y,z,t\right)}{dx}V=\frac{q\eta }{4c\rho {\sqrt{a\pi }}^{3}}\int_{t0}^{t}\frac{x+V\tau }{2a\tau {\sqrt{\tau }}^{3}}\cdot exp\left[-\frac{{\left(x+V\tau \right)}^{2}+y^{2}}{4a\pi }\right]\cdot \sum_{-\infty }^{\infty }exp\left[-\frac{{\left(z+2n\delta \right)}^{2}}{4a\pi }\right]d\tau$$

In the calculations, the thermophysical characteristics of the material were assumed constant, as an average value in the temperature range of 100–600 °C.

**Figure 18:**
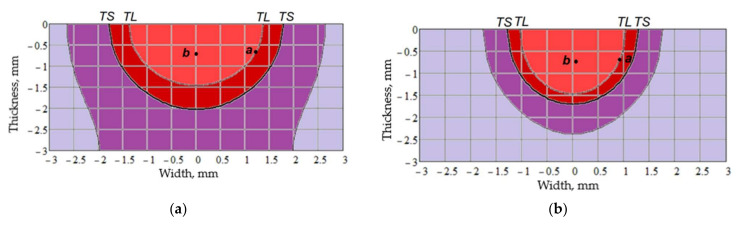
Temperature distribution in the cross section during welding: (**a**) MIG; (**b**) EBW.

**Figure 19:**
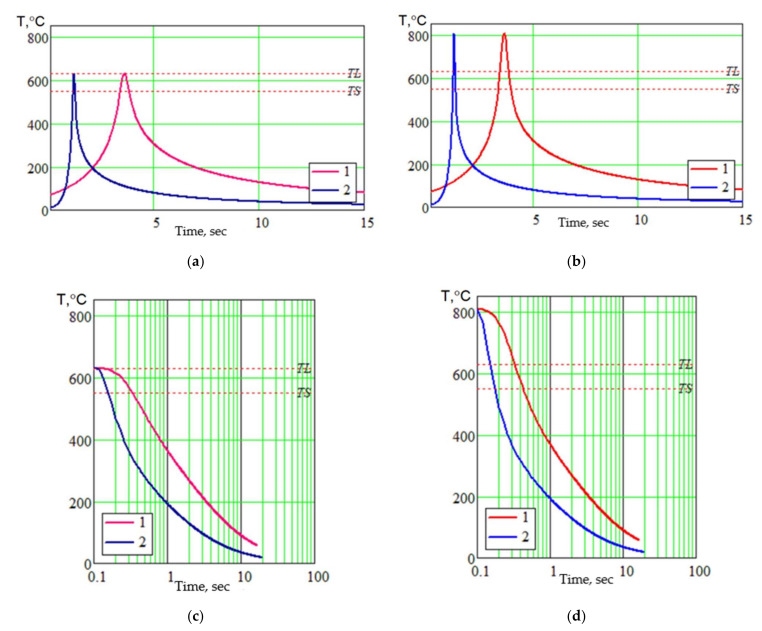

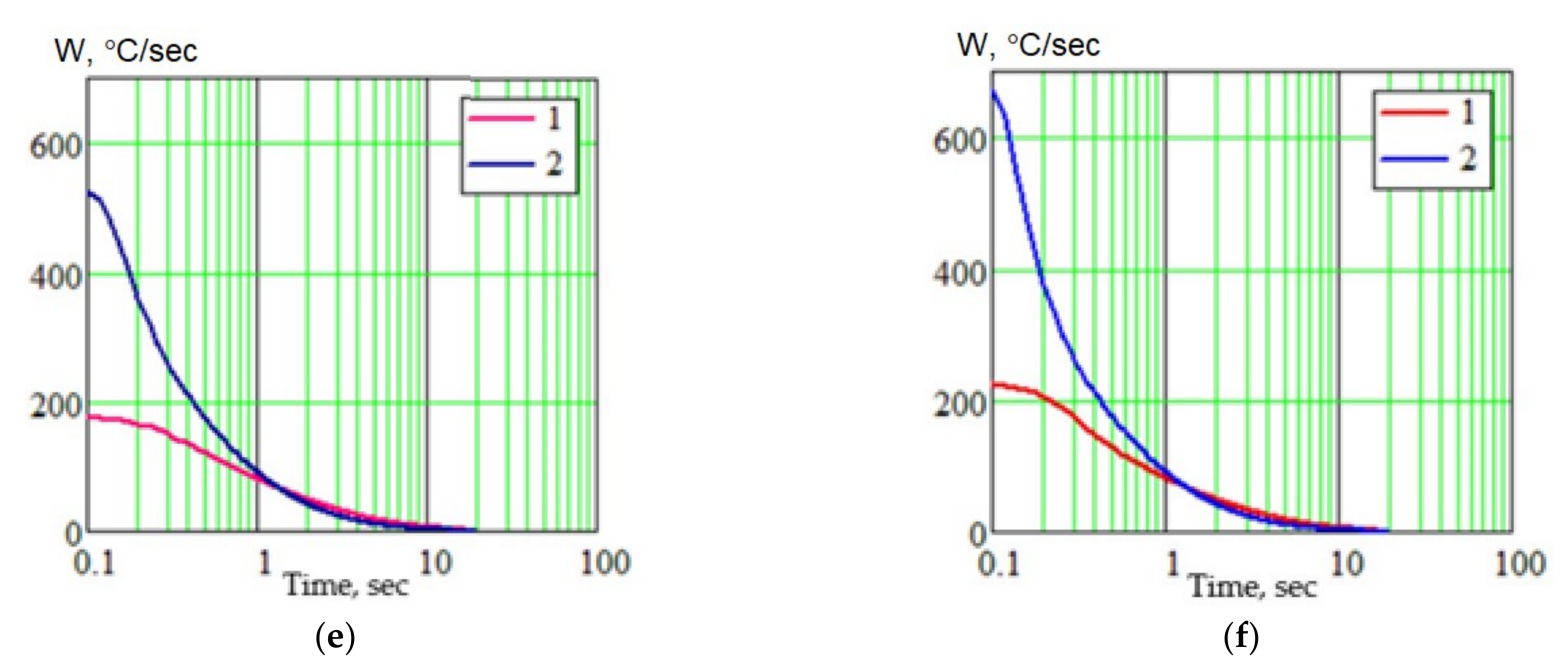
Calculation results of thermal cycle parameters for MIG (1) and EBW (2) welding for the fusion zone (**a**,**c**,**e**) and weld metal (**b**,**d**,**f**): (**a**,**b**) thermal cycle; (**c**,**d**) cooling curves; (**e**,**f**) instant cooling rates.

The application of this model makes it possible to calculate the temperature distribution in the welded joint, temperature-time modes, and instant cooling rates for different sections of the welded joint. The application software MathCad was used for the calculations. As we can see in Figure 18 the calculated isothermal lines in the cross-section of the welded joint made by MIG welding (Figure 18a) and EBW welding (Figure 18b) are presented. Isotherms are corresponding to the crystallization start temperature TL, the crystallization end temperature TS and the temperature above which structural increments (grain growth, dissolution of strengthening phases, coagulation of phases) happen in the base metal. Calculations have shown that the extent of the fusion zone at EBW is almost 2 times less than at MIG welding. For the metal heated to the temperature of the beginning of crystallization TL (point a in Figure 18) at MIG and EBW welding was calculated the thermal cycle, both metal cooling curves and instant cooling rate were built. Similar calculations were made for the weld metal (point b in Figure 18). These results are presented in Figure 19.

As the calculations show, the residence time of the metal in the fusion zone and the weld metal in the liquid state at EBW is significantly less than at MIG welding (almost 2.5). The same instant cooling rate of the alloy zone metal and weld metal during crystallization at EBW is significantly higher than the cooling rate at MIG welding.

## 4. Discussion

According to the results of the studies, the characteristics of the strengthening phases formation were revealed in the metal of the Al–MG–LI alloy with zirconium additives. In particular, it was found that in the metal without TVT there is an allocation of stable strengthening phases and one metastable phase. The nature of the allocation of strengthening phases in the metal with TVT and without TVT differs significantly. The content of chemical elements insignificantly changes in the released phases depending on temperature. In particular, after the standard heat treatment T6 the hardening stable phases Al_2_LiMg and Al_3_Mg_2_ and the metastable phase Al_3_Li are allocated.

From the literature data [13,20,21], it is known that in the 1420 alloy directly after quenching in the water there is a certain amount of dispersed precipitation of the intermediate hardening phase (Al_3_Li). The decomposition of the supersaturated solid solution in 1420 alloy occurs according to the scheme: α→ (Al3Li)→ S1(AL2MgLi). The phase (Al_3_Li) is an intermediate unstable phase that has a cubic Li_2_ lattice whose parameter is close to the lattice parameter of aluminum; thus, the precipitates (Al_3_Li) formed in the solid solution during cooling from the quenching temperature and during subsequent aging are completely coherent with the matrix and remain so in a wide range of aging temperatures. The second phase S1—the phase having a complex cubic lattice, is formed from—excretions or is isolated directly from the solid solution and is partially coherent with the matrix.

Accordingly, thermodynamic calculations correlate with the literature data. In addition, the research shows that thermal vacuum treatment with subsequent heat treatment contributes to the alignment of the chemical elements along the grain body and fixation of homogeneous solid solution, which characterizes the uniform isolation of fine-dispersed strengthening phases, also confirmed by metallographic studies. The results of metallographic studies of welded joints showed not only the peculiarity of fine-dispersed phases allocation but also the nature of the formation and distribution of pores in the metal with TVT and without TVT. As known from [11,13,16], pore formation depends on hydrogen concentration. The presence of a large concentration of hydrogen is the main cause of porosity in the weld metal.

The X-ray phase composition data correlate with the calculated results of thermodynamic analysis and show that TVT followed by quenching and aging has no effect on the phase composition of the alloy.

In addition to the effect of hydrogen concentration in the base metal of aluminum alloys, both the ratio of alloying elements and the welding technology largely influence the quality of the weld, which determines the properties of the welded joint.

Research on the influence of TVT and welding technology on the formation of welded joints showed that pores form in MIG welds regardless of TVT. Pores were not detected in EBW welding. In addition, it was found that two main phases (Al_6_Mn and Al_3_Mg_2_) are formed in the temperature range of the beginning and end of crystallization of the alloy in the welds made by MIG. It was determined that the formation of these phases occurs not only in the weld at the final stage of crystallization but also in the fusion zone. The formation of phases in the two-phase solid–liquid fusion zone can also initiate the formation of pores. This fact can be correlated with the literature data, from which it follows that, when the crystallization front moves, gas accumulates in a narrow boundary layer of liquid metal. At a high saturation of the boundary layer of the liquid metal with gas, its concentration exceeds the standard solubility. During aluminum crystallization, the hydrogen available in it is completely fixed in the solid metal and then redistributed between the solid and liquid metal according to the laws of diffusion. In this case, the process controlling the transition of hydrogen to liquid becomes the diffusion of hydrogen in the solid phase [11,13].

The solubility of hydrogen in liquid aluminum is affected by the alloying elements. In addition, the introduction of magnesium into the alloy reduces the hydrogen diffusion coefficient. Therefore, it should be taken into account that it is MIG welding that makes use of welding additive material with Mg, as opposed to EBW, which is carried out without additive metal and in a vacuum.

The results of microhardness determination showed a general tendency of decreasing hardness in the weld in contrast to the base metal. However, in MIG welding after TVT, a more decreased hardness in the weld relative to the base metal was observed. Whereas a different tendency was observed at EBW. This can be explained as follows. EBW is mainly designed to remove excessive hydrogen. Since hydrogen with aluminum forms an embedding solid solution, this means that its percentage should have an effect on hardness. EBW is carried out in a vacuum and the alloy is degassed when the edges of the base material melt. In the case of welding the base material, the difference between the percentage of dissolved hydrogen in the base metal and in the weld will be higher than in the case of welding the base material after EBW, consequently, the difference between hardness will be greater.

Calculations of thermal cycles of welding showed the residence time of the metal in the fusion zone and the weld metal in the liquid state, which at EBW is much less than at MIG welding (almost 2.5). Given the short residence time of the weld metal in the liquid state and high cooling rates at the time of crystallization at EBW, a homogeneous solid solution will be formed in the weld, which is confirmed by metallographic studies. The absence of pores in the fusion zone and weld metal at EBW is caused by processes of degassing of metal and a rigid thermal cycle of welding. At MIG welding, the formation of pores and large inclusions in the fusion zone are connected with the peculiarity of formation of strengthening phases in the liquid–solid state, its large extent, and a longer stay of metal in this state.

## 5. Conclusions

Based on the results of the research on the interaction of base material, thermal vacuum treatment, and welding technology on pore formation in welded joints of Al–MG–LI alloys with zirconium additives, the following conclusions can be made.

Thermodynamic calculations made it possible to determine the features of the formation of strengthening phases in the base metal with and without TVT. It was revealed that, in the metal after standard heat treatment without TVT, the formation of strengthening phases Al_2_LiMg, Al_3_Mg_2_, Al_3_Li is characteristic. In addition, calculations have shown that TVT with subsequent quenching and aging has no effect on the phase composition of the alloy, but the nature of the evolution of fine-dispersed strengthening phases becomes uniform.

The study of microstructure and calculations of thermal cycles of welded joints established that the MIG technology (for metal with TVT and without TVT) is characterized by a large extent of the fusion zone, the high residence time of metal in the fusion zone, and weld metal in the liquid state, pore formation. As the weld pool cools, hydrogen atoms tend to be released due to a sharp decrease of solubility. However, hydrogen meets and combines with other hydrogen atoms, crystallization centers, and impurities in the metal recombine into molecules and form gas bubbles. These bubbles float as long as the ductility of the surrounding metal allows. Gas bubbles that do not resurface in time remain in the metal after it has crystallized, forming porosity, whereas in EBW welding, the opposite tendency is observed with the absence of pores.

A more uniform distribution of hardness was revealed in the welded joints made by EBW. However, application of TVT before welding leads to a more uniform hardness of the weld metal, in both cases of welding.

EBW technology prevents pore formation and makes it possible to produce welded joints of 1420 Al alloys of the required quality.

## Figures and Tables

**Table 1:** Chemical composition of AlMg-6 welding additive wire.

	Chemical Element Content, at. %				
	Al	Mg	Mn	Ti	Si
GOST 7871-2019	Base	5.8–6.8	0.5–08	0.1–0.2	to 0.4
For calculation	Base	6.6	0.8	0.2	0.3

Argon arc welding modes: welding current—235 A; shielding gas flow—12 L/min; wire feed speed—0.24…0.27 m/min. Welding was carried out on a welding machine BlueWeld MIGMAG 500S. Modes of EBW: accelerating voltage Uu = 20 kV, welding current Iw = 130 mA, welding speed Vw = 55 m/h. Welding was carried out on the machine AELTK-343. Workpieces made of aluminum wrought heat-hardenable 1420 alloy to create welded constructions before welding are subjected to mandatory standard heat treatment (marking T6) according to the mode: (1) hardening (450 ± 15 °C with cooling in the air); (2) artificial aging (120 ± 15 °C for a time of 5 h, cooling in the air).

**Table 2:** Chemical composition of the 1420 Al–MG–LI alloy.

	Chemical Element Content, at. %						
	Al	Mg	Li	Zr	Si	Fe	Mn
OST 1-90048-90-90	Base	5–6	1.9–2.3	0.09–0.15	0.1–0.3	to 0.3	to 0.3
For the calculation	Base	5.5	2.1	0.12	0.2	-	-

**Table 3:** Calculated phase composition of 1420 Al–MG–LI alloy after heat treatment.

Phases	% Phase in the Alloy	Concentration of Chemical Elements, at. %				
		Al	Li	Mg	Si	Zr
Al	The other content	97.86	0.59	1.54	<0.01	<0.01
Al_3_Zr (AL3M_DO23)	0.54	75.0	0.0615	-	-	24.94
Mg_2_Si (MG2SI)	3.37	-	-	66.67	33.33	-
Al_2_LiMg (TAU_ALLIMG)	21.37	53.0	33.0	14.0	-	-
Al_3_Mg_2_ (AL3MG2)	0.13	61.5	2.94	35.56	-	-
Al_3_Li (AL3M_L12)	8.56	76.14	23.76	0.0937	-	<0.01

**Table 4:** Results of diffractograms for the 1420 alloy after standard heat treatment T6 (1) and after TWO (2).

№ Peak	№ Sample	Pos. (°2 Th.)	D-Spacing (A)	Matched by № Substance in the Database «ICDD PDF-2»
1	1	382,694	234,998	01-089-2837
	2	382,494	235,116	
2	1	444,547	203,630	01-089-2837; 03-065-7533
	2	444,477	203,661	
3	1	647,267	143,904	01-089-2837; 03-065-5657; 03-065-7533
	2	646,903	143,976	
4	1	776,824	122,823	01-089-2837; 03-065-7533
	2	776,979	122,802	
5	1	819,205	117,507	01-089-2837
	2	818,811	117,553	

**Table 5:** Calculated phase composition of weld metal with AlMg-6 welding additive wire.

Phases	% Phase in the Alloy	Chemical Element Content, at. %				
		Al	Mg	Mn	Si	Ti
Al	The rest	99.65	0.35	<0.01	<0.01	<0.01
Al_3_Ti (AL3M_DO22)	0.45	75.0	-	<0.01	-	25
Al_6_Mn (AL6MN)	2.74	85.7	<0.01	14.29	-	-
Mg_2_Si (MG2SI)	1.15	-	66.67	-	33.33	-
Al_3_Mg_2_ (AL3MG2)	16.27	61.3	38.5	-	-	-

**Table 6:** Microhardness of the weld metal, when welding according to different options.

	Hardness HV 0.05 MPa/mm^2^			
	**MIG**		**EBW**	
	Without TVT *	With TBO *	Without TVT *	With TBO *
Average Value	993.7	823.1	859.0	850.5
Minimum value	783.4	585.8	698.7	558.8
Maximum value	1188.4	1084.9	1088,3	1011.9
σ—Standard deviation	103.2	80.5	85.3	95.4
υ—Variation coefficient (%)	10.3	9.7	9.9	11.2
% Hardening relative to the parent metal	22.7	35.0	21.8	30.1

* Condition of the initial alloy.

## Data Availability

Data is contained within the article.
